# A systemic pharmacovigilance assessment of ophthalmic atropine: signal detection and clinical prioritization from the FAERS database

**DOI:** 10.3389/fmed.2026.1798521

**Published:** 2026-03-18

**Authors:** Zihang Xu, Jizhou Liang, Wenting Cai, Yunhong Luo, Feng Liang, Ruoyi Lin, Chengyu Hu, Tu Su, Yan Wu, Jia He, Jing Yu

**Affiliations:** 1 Department of Ophthalmology, Shanghai Tenth People’s Hospital, School of Medicine, Tongji University, Shanghai, China; 2 Department of Health Statistics, Faculty of Health Service, Naval Medical University, Shanghai, China; 3 Department of Ophthalmology, Shanghai Pudong Hospital, Fudan University Pudong Medical Center, Shanghai, China; 4 Department of Ophthalmology, The Third People’s Hospital of Bengbu, Bengbu, China

**Keywords:** adverse drug reaction, adverse reaction signal, data mining, FAERS database, ophthalmic atropine

## Abstract

**Purpose:**

To comprehensively and systematically explore adverse drug reactions (ADRs) associated with ophthalmic atropine, providing evidence-based safety references for clinical medication practices.

**Methods:**

Signal detection for ophthalmic atropine-associated ADRs was conducted using the Information Component (IC) and Reporting Odds Ratio (ROR) methods, analyzing data from the inception of the FDA Adverse Event Reporting System (FAERS) database through the second quarter of 2025.

**Results:**

The FAERS database contained 425 reports of ophthalmic atropine-associated ADRs, with 83 positive signals detected. These signals primarily involved eye disorders, nervous system disorders, injury, poisoning and procedural complications, and infections and infestations. Endophthalmitis (*n* = 74, IC_025_ = 6.62, ROR_025_ = 101.90) was the most frequent and strongest ADR signal detected. Other high-intensity ADR signals were choroiditis (IC_025_ = 6.05, ROR_025_ = 69.26), intraocular pressure increased (IC_025_ = 5.77, ROR_025_ = 56.93), visual acuity reduced (IC_025_ = 5.46, ROR_025_ = 45.74), mydriasis (IC_025_ = 5.36, ROR_025_ = 43.34), and uveitis (IC_025_ = 5.36, ROR_025_ = 42.93). Additionally, eye pain (*n* = 64, IC_025_ = 4.99, ROR_025_ = 32.90) represented another frequently reported ADR after endophthalmitis. Of the preferred terms, 81.93% were assigned a grade of weak clinical priority, with the remainder (18.07%) falling into the moderate category.

**Conclusion:**

Ophthalmic atropine demonstrates potential ADR burdens in ocular systems, necessitating heightened clinical vigilance and prompt risk mitigation strategies to ensure medication safety. It should be noted that these findings represent safety signals from a spontaneous reporting database, not incidence estimates or proof of causality.

## 1 Introduction

Atropine, a non-selective muscarinic antagonist, has been used in ophthalmology for decades (1). Its ocular effects support a range of clinical applications, including routine fundus examinations, uveitis management, amblyopia therapy. In recent years, low-concentration atropine has gained widespread use for myopia control in children, particularly in East Asia (2, 3).

Although topical atropine is generally considered safer than systemic administration, it is not free from adverse effects. Ocular adverse reactions associated with it range from local anticholinergic effects (e.g., blurred vision, photophobia) to hypersensitivity reactions (e.g., conjunctival hyperemia, eyelid edema) (4, 5), as well as potential systemic anticholinergic effects following absorption (6).

Nevertheless, the full safety profile of ophthalmic atropine remains incompletely understood particularly regarding rare but serious adverse events (AEs), such as severe systemic anticholinergic reactions (e.g., tachycardia) and uncommon ophthalmic complications (e.g., acute angle-closure glaucoma) (4–7). Pre-marketing clinical trials are limited by small sample sizes, short follow-up, and exclusion of complex patients, making them underpowered to detect rare events. Current evidence largely relies on sporadic case reports (8, 9), which lack denominators for quantitative risk assessment. Systematic analysis of post-marketing real-world data is therefore essential. The FDA Adverse Event Reporting System (FAERS), a global pharmacovigilance database, enables detection of such signals through disproportionality analysis. Given atropine’s expanding use across diverse populations, including pediatric myopia control and adult uveitis management, FAERS is particularly suited for generating hypotheses about potential safety concerns warranting further investigation.

Given its expanding role in myopia control and its long-standing use in other ocular conditions, a clearer understanding of its adverse reaction profile is necessary to support clinical decision-making and patient monitoring. This study therefore aimed to perform a comprehensive data mining analysis of the FAERS database to detect and characterize potential adverse reaction signals linked to ophthalmic atropine use, thereby providing evidence-based safety information for clinical practice.

## 2 Materials and methods

### 2.1 Data sources

This real-world, retrospective study conducted a disproportionality analysis using the FAERS database, covering data from its inception through Q2 2025 (the most recent update available at study initiation). FAERS, a prototypical spontaneous reporting system, aggregates adverse event reports, medication error reports, and product quality complaints submitted by healthcare professionals, consumers, and manufacturers.

### 2.2 Procedures

The data processing and analysis in this study followed a structured stepwise approach. Essential variables, including PRIMARYID, CASEID, SEX, DRUGNAME, ROLE_COD, and Preferred Terms (PTs), were extracted from the respective data files within the FAERS database.

Step 1: Duplicate report removal. Since the database collects reports from various sources, duplicate entries are inevitable. To minimize the risk of both false-positive and false-negative signals, we first identified and eliminated duplicate reports. This was accomplished using a standardized variable-matching approach, a method commonly adopted by regulatory agencies such as the UK Medicines and Healthcare products Regulatory Agency (MHRA) and the Danish Health and Medicines Authority (DHMA) (10). The variable matching approach identifies duplicate reports by comparing key fields between records. When these predefined variables (such as report identifiers, patient demographics (e.g., sex, birth date), and suspect drug information) are identical, the reports are considered duplicates. In accordance with FDA recommendations, only the most recent version of such duplicate reports was retained for analysis. In this study, PRIMARYID, CASEID, CASEVERSION, and FDA_DT^1^ were selected as the essential variables for duplicate detection. The procedure was performed as selecting the latest FDA_DT when the PRIMARYIDs were the same, while selecting the largest CASEID and CASEVERSION when the FDA_DT and the PRIMARYID were the same.

Step 2: Selection of suspect drugs. Drugs listed in the reports were classified into four categories based on their reported role: Primary Suspect (PS), Secondary Suspect (SS), Concomitant (C), and Interacting (I). We only included the records with drug patterns of PS and SS, in order to avoid confounding noise introduced by reports involving interacting or concomitant medications, and to obtain an independent safety profile associated with ophthalmic atropine use. This approach has also been adopted by the Uppsala Monitoring Centre of the World Health Organization.

Step 3: Identification of atropine-related reports. The FAERS database contains two medication-related variables: DRUGNAME and PROD_AI. To ensure comprehensive identification of the target medications, we queried both generic names and brand names across these two fields. Meanwhile, we only included the records of atropine administration through “ocular administration” in the analysis, and these records were determined based on the “ROUTE” variable in the drug files. To ensure that only confirmed ophthalmic administrations were analyzed, reports with missing or unspecified route information were excluded.

Step 4: Adverse event coding and data extraction. According to MedDRA version 28.0, all adverse events in the study were coded in the PT. When data were available, relevant clinical and reporting characteristics were extracted and analyzed, including patient sex, age, reporting country, indication, clinical outcome, and year of report. Missing or unspecified values for these variables were categorized as “Missing or unknown” and reported separately in the descriptive tables.

**Figure 1:**
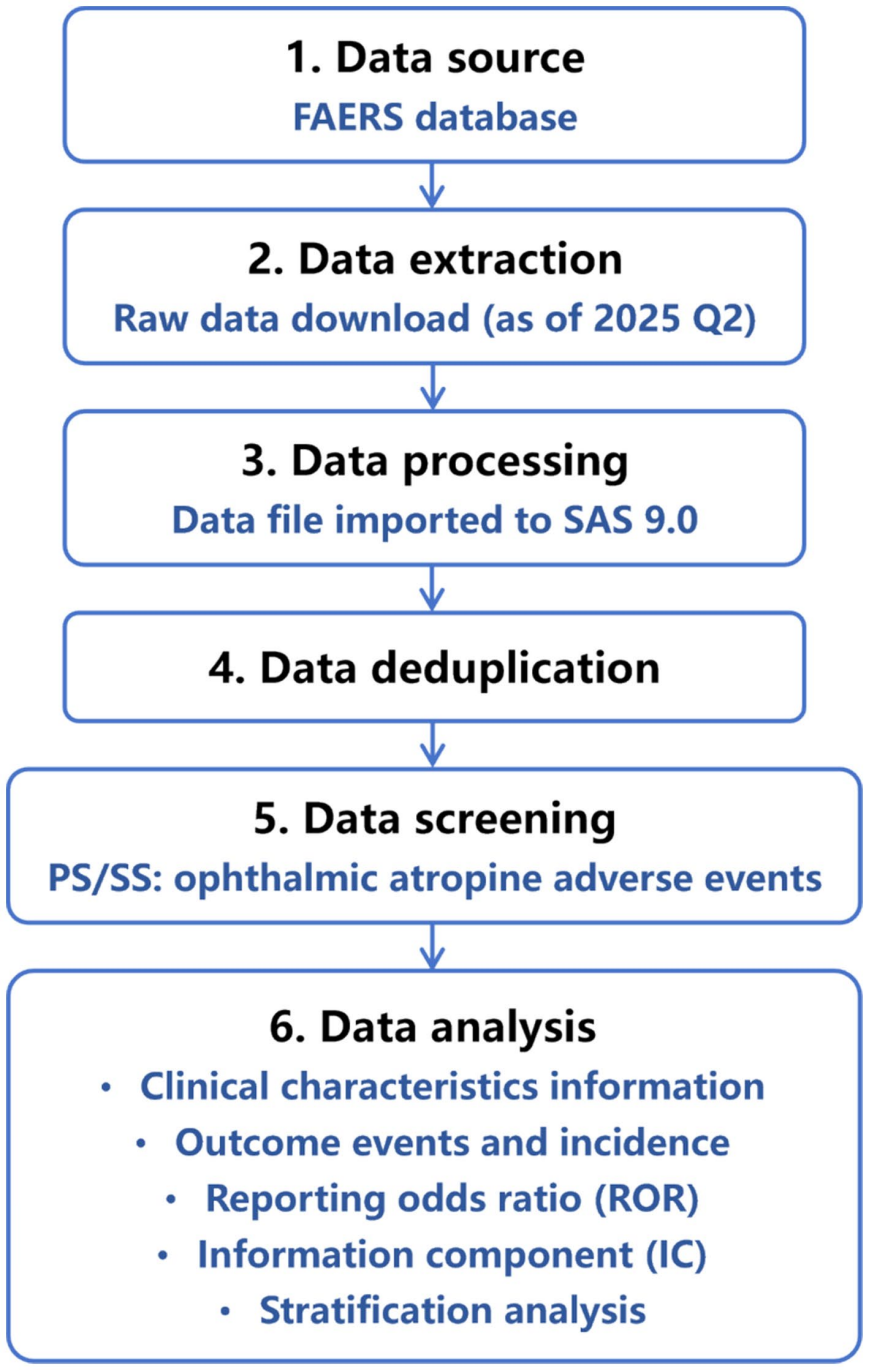
Multistep process of data extraction, processing, and analysis from the Food and Drug Administration adverse event reporting system database.

The overall workflow for data extraction, processing, and analysis is presented in Figure 1: FAERS raw data were downloaded, processed, and filtered for reports listing ophthalmic atropine as a suspect drug. Clinical characteristics were extracted, followed by disproportionality analysis using ROR and IC, with stratification to adjust for confounders.

### 2.3 Statistical analysis

Reporting odds ratio (ROR) and Bayesian confidence propagation neural networks of information components (IC) are two specific indices that are calculated to detect potential associations between ophthalmic atropine and AEs (11). For the sake of robustness, statistical shrinkage transformation was performed. The calculation formulas for ROR and IC after transformation are as follows (12, 13):

$$\mathrm{ROR}=\frac{N_{\text{observed}}+0.5}{N_{\text{expected}}+0.5}$$

$$\mathrm{IC}=\log_{2}\frac{N_{\text{observed}}+0.5}{N_{\text{expected}}+0.5}$$

$$N_{\text{expected}}=\frac{N_{\text{drug}}*N_{\text{vente}}}{N_{\text{total}}}$$

where *N*_observed_ is the observed number of records of target drugAEs, *N*_expected_ is the expected number of records of target drugAEs, *N*_drug_ is the total number of records of the target drug, *N*_event_ is the total number of records of target AEs, and *N*_total_ is the total number of records in the whole database.

The calculation is based on two-by-two contingency tables. The lower limit of the 95% confidence interval for both ROR (ROR_025_) and IC (IC_025_) are criteria for a significant signal. If ROR_025_ is greater than one or IC_025_ exceeding zero, and with at least three records, it would be considered statistically significant and deemed a potential signal. The calculation formulas are as follows (12):

$$\mathrm{ROR}95\%\mathrm{CI}=e^{\ln(\mathrm{ROR})\pm 1.96\sqrt{\frac{1}{a}+\frac{1}{b}+\frac{1}{c}+\frac{1}{d}}}$$

$$IC_{025}=\mathrm{IC}-3.3\times {\left(N_{\text{observed}}+0.5\right)}^{-0.5}-2\times {\left(N_{\text{observed}}+0.5\right)}^{-1.5}$$

$$IC_{975}=\mathrm{IC}+2.4\times {\left(N_{\text{observed}}+0.5\right)}^{-0.5}-0.5\times {\left(N_{\text{observed}}+0.5\right)}^{-1.5}$$

ROR and IC were calculated to explore the spectrum of systemic adverse reactions associated with ophthalmic atropine. All analyses were performed using SAS version 9.4 (SAS Institute Inc., Cary, NC, United States).

### 2.4 Clinical prioritization of signals

To identify safety signals that warrant heightened attention in routine pharmacovigilance activities beyond mere statistical association, a clinical prioritization framework was employed. This approach is grounded in the Designated Medical Event (DME) and Important Medical Event (IME) lists established by the European Medicines Agency, which catalog adverse events of special concern regardless of their frequency. Building upon this, a semiquantitative scoring system was applied to rank signals of significant disproportionality based on their potential clinical relevance (14, 15). It was designed to support hypothesis generation in pharmacovigilance research and has been applied in previous studies (15).

Signals were evaluated across five key dimensions: (1) the absolute number of reports; (2) the strength of association, as indicated by the lower limit of the 95% confidence interval for the Reporting Odds Ratio (ROR_025_); (3) the seriousness of outcome, measured by the proportion of reports with a fatal result; (4) designation as a DME or IME; and (5) biological and clinical plausibility. A composite score was calculated by summing the points from each dimension. Based on this total, signals were categorized into three tiers of clinical priority: a score of 0–4 indicated weak priority, 5–7 indicated moderate priority, and 8–10 indicated strong priority. The complete scoring methodology and criteria are detailed in Table 1.

**Table 1:** A rating scale assessing clinical priority of disproportionality signals.

Assessment items	2 points	1 point	0 point
Number of target events	>50	10–50	<10
ROR	>5	2–5	1–2
Mortality proportion	>50%	25–50%	<25%
IMEs or DMEs	DME	IME	None
Relevant evidence evaluation	++	+	−

Mortality proportion: percentage of cases in which death was reported as an outcome in the overall cases report for a particular adverse event. IMEs and DMEs are developed and updated by EMA (European Medicines Agency, 2022). ++: AEs are mainly from the FDA Prescribing Information, the Summary of Product Characteristics posted by the MHRA, Phase 2/3 RCTs, or systematic reviews, with biological plausibility. +: AEs are mainly from other clinical trials, observational studies, or case reports/series with potential biological plausibility. –: AEs only emerging from disproportionality analyses. AEs, Adverse Events; DMEs, Designated Medical Events; IMEs, Important Medical Events; MHRA, Medicine and Healthcare Products Regulatory Agency; RCTs, Randomized Controlled Trials; ROR, Reporting odds ratio.

## 3 Results

### 3.1 Descriptive analysis

A total of 1,511 reports of AEs associated with ophthalmic atropine were included in the final analysis. The demographic and reporting characteristics are detailed in Table 2. The reports were more frequent in males (49.57%) than in females (36.33%), and adults aged 19 to 64 years accounted for the majority (53.21%). Iridocyclitis was the most commonly reported indication (8.07%). Geographically, the highest number of reports originated from Canada (37.13%), followed by France (20.32%) and the United States (17.27%). Regarding clinical outcomes, the most frequently reported category was “Other Serious (Important Medical Event)^2^” (58.70%), followed by hospitalization (26.54%). Death and life-threatening events were reported in 2.78 and 1.59% of cases, respectively. It should be noted that data were missing or unknown for a proportion of reports across multiple variables, which may introduce potential reporting bias and should be considered when interpreting the findings.

**Table 2:** Characteristics of patients with ophthalmic atropine-associated adverse events (*n* = 1,511).

Characteristics	Ophthalmic atropine induced AEs *n* (%)
Sex	
Male	749 (49.57)
Female	549 (36.33)
Missing or unknown	213 (14.10)
Age	
≤3	119 (7.88)
4–18	95 (6.29)
19–64	804 (53.21)
≥65	132 (8.74)
Missing or unknown	361 (23.89)
Indications	
Iridocyclitis	122 (8.07)
Product used for unknown indication	115 (7.61)
Ophthalmological examination	36 (2.38)
Postoperative care	27 (1.79)
Fundoscopy	19 (1.26)
Mydriasis	16 (1.06)
Myopia	16 (1.06)
Strabismus	12 (0.79)
Toxicity to various agents	10 (0.66)
Cataract congenital	9 (0.6)
Endophthalmitis	9 (0.6)
Iritis	9 (0.6)
Preoperative care	9 (0.6)
Salivary hypersecretion	9 (0.6)
Uveitis	8 (0.53)
Others	142 (9.39)
Missing or unknown	943 (62.40)
Reporting countries	
Canada	561 (37.13)
France	307 (20.32)
America	261 (17.27)
United Kingdom	53 (3.51)
Brazil	51 (3.38)
Germany	44 (2.91)
Italy	40 (2.65)
Thailand	25 (1.65)
Other countries	127 (8.41)
Missing or unknown	42 (2.78)
Outcome	
Other serious (important medical event)	887 (58.70)
Hospitalization – initial or prolonged	401 (26.54)
Disability	82 (5.43)
Death	42 (2.78)
Life-threatening	24 (1.59)
Missing or unknown	75 (4.96)

AEs, adverse events; n, number of cases.

### 3.2 Disproportionality analysis

As detailed in Table 3, a disproportionality analysis at the System Organ Classes (SOCs) level was conducted for ophthalmic atropine using the FAERS database. At the SOC level, only one SOC demonstrated a statistically significant positive signal: Eye Disorders (*n* = 476, ROR 20.86, 95% CI: 18.72–23.26, IC 4.38, 95% CI: 4.23–4.49). In contrast, the ratios of all other SOC reports showed no statistically significant differences. This indicates that in this analysis, no significant imbalance signals were detected in the non-ocular system organ categories.

**Table 3:** Signal strength of reports of ophthalmic atropine at the system organ class (SOC) level in the FAERS database.

System organ class (SOC)	Ophthalmic atropine cases reporting SOC	ROR (95% two-sided CI)
Eye disorders *	476	20.86 (18.72–23.26)
Blood and lymphatic system disorders	2	0.09 (0.02–0.37)
Cardiac disorders	33	1.02 (0.72–1.44)
Congenital, familial and genetic disorders	1	0.39 (0.05–2.77)
Ear and labyrinth disorders	3	0.56 (0.18–1.75)
Gastrointestinal disorders	76	0.55 (0.44–0.69)
General disorders and administration site conditions	168	0.72 (0.61–0.84)
Hepatobiliary disorders	1	0.10 (0.01–0.69)
Immune system disorders	9	0.46 (0.24–0.89)
Infections and infestations	112	1.17 (0.96–1.42)
Injury, poisoning and procedural complications	152	1.09 (0.92–1.29)
Investigations	96	0.88 (0.72–1.09)
Metabolism and nutrition disorders	23	0.62 (0.41–0.94)
Musculoskeletal and connective tissue disorders	9	0.08 (0.04–0.16)
Neoplasms benign, malignant and unspecified (incl cysts and polyps)	6	0.20 (0.09–0.45)
Nervous system disorders	110	1.01 (0.83–1.22)
Product issues	14	1.28 (0.76–2.17)
Psychiatric disorders	67	0.91 (0.71–1.16)
Renal and urinary disorders	24	0.71 (0.48–1.07)
Respiratory, thoracic and mediastinal disorders	17	0.22 (0.14–0.36)
Skin and subcutaneous tissue disorders	63	0.80 (0.62–1.03)
Surgical and medical procedures	2	0.16 (0.04–0.63)
Vascular disorders	25	0.81 (0.55–1.20)

* Indicates statistically significant signals in the algorithm. ROR, reporting odds ratio; CI, confidence interval.

**Figure 2:**
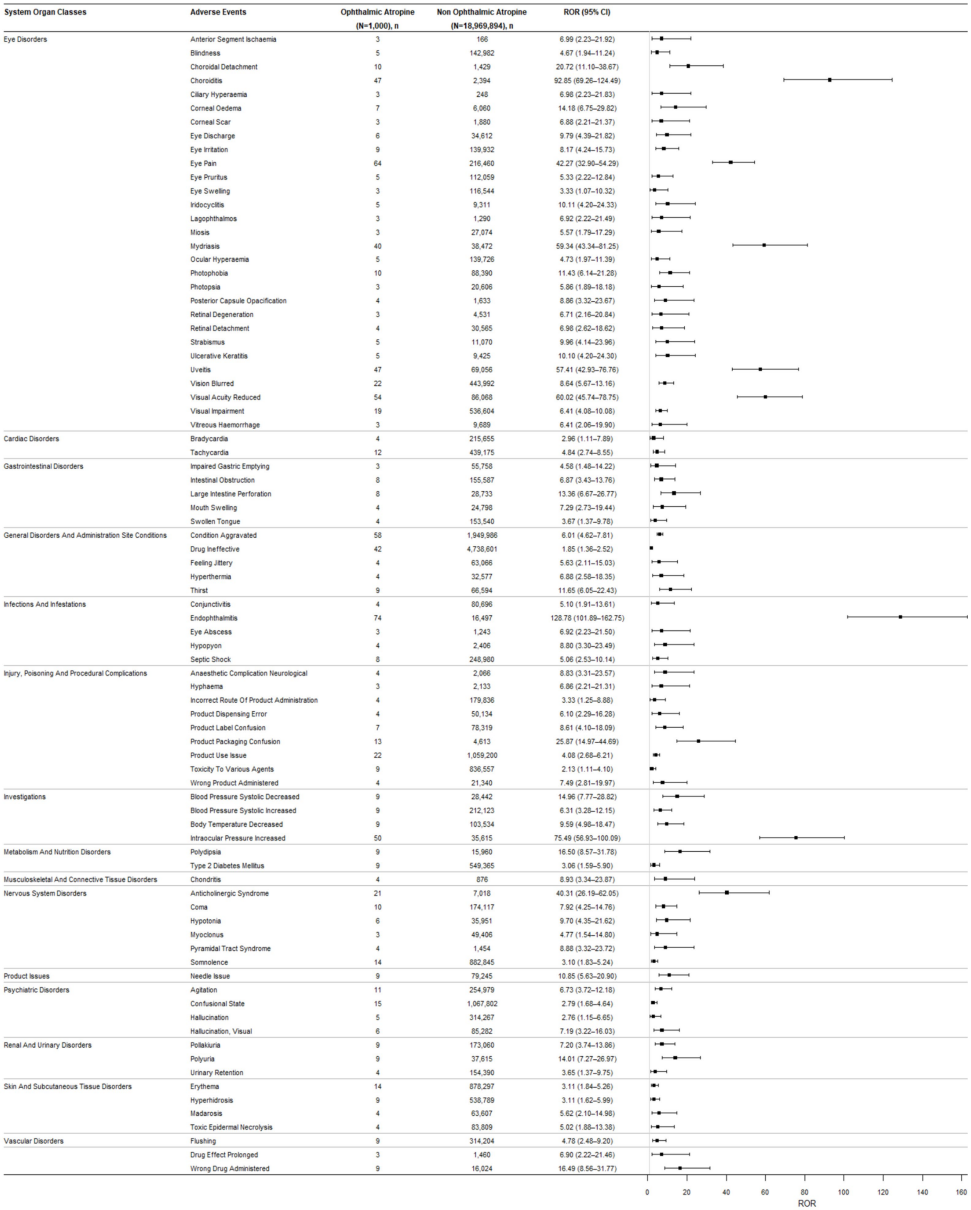
Reporting odds ratios (ROR) with 95% CI for all positive ophthalmic atropine-associated adverse events (AEs). *N*, number of cases of total AEs associated with the ophthalmic atropine; *n*, number of cases with suspected AEs associated with the ophthalmic atropine; ROR, reporting odds ratio; CI, confidence interval; AEs, adverse events.

At the more granular preferred terms (PTs) level, a total of 83 positive signals associated with ophthalmic atropine were detected across 16 System Organ Classes (Figure 2). The signal strength ranking for these adverse events is presented in Supplementary Table S1. Endophthalmitis represented both the most frequently reported and the strongest individual adverse reaction signal identified (*n* = 74, IC_025_ = 6.62, ROR_025_ = 101.90).

In the SOC of Eye disorders, notable high-strength signals within this class included choroiditis (IC_025_ = 6.05, ROR_025_ = 69.26), visual acuity reduced (IC_025_ = 5.46, ROR_025_ = 45.74), mydriasis (IC_025_ = 5.36, ROR_025_ = 43.34), and uveitis (IC_025_ = 5.36, ROR_025_ = 42.93). Eye pain (*n* = 64, IC_025_ = 4.99, ROR_025_ = 32.90) was another frequently reported event.

Strong signals were also detected in other SOCs. Under Investigations, intraocular pressure increased (IC_025_ = 5.77, ROR_025_ = 56.93) was a prominent signal. Significant associations were found in Nervous system disorders (e.g., anticholinergic syndrome, IC_025_ = 4.6, ROR_025_ = 26.19) and Injury, poisoning and procedural complications (e.g., product packaging confusion, IC_025_ = 3.75, ROR_025_ = 14.97). Notably, several positive signals pertained to systemic anticholinergic effects beyond the eye, such as tachycardia (Cardiac disorders, IC_025_ = 1.3, ROR_025_ = 2.74), thirst (General disorders, IC_025_ = 2.4, ROR_025_ = 6.05), polydipsia (Metabolism disorders, IC_025_ = 2.91, ROR_025_ = 8.57), and agitation (Psychiatric disorders, IC_025_ = 1.73, ROR_025_ = 3.72).

### 3.3 Clinical prioritization of the disproportionality signals

As presented in Table 4, among the 83 disproportionality signals that reached statistical significance, 26 (31.33%) were classified as IMEs, while two (2.41%) were DMEs: blindness (ROR 4.67, 95% CI: 1.94–11.24) and toxic epidermal necrolysis (ROR 5.02, 95% CI: 1.88–13.38). Based on the clinical priority scoring system which integrates signal strength, clinical relevance, and supporting evidence, 68 (81.93%), 15 (18.07%), and 0 signals were graded as weak, moderate, and strong clinical priorities, respectively. The absence of strong priority signals suggests that no adverse event met the highest threshold for clinical concern. Four disproportionality signals attained the highest priority score of 6 within the moderate priority category: visual acuity reduced, eye pain, anticholinergic syndrome, and coma. In the evaluation of supporting evidence, 34 signals demonstrated a strong level of evidence, denoted as “++,” which was defined as adverse events primarily documented in FDA prescribing information, the Summary of Product Characteristics posted by the MHRA, Phase 2/3 randomized controlled trials (RCTs), or systematic reviews, with biological plausibility.

**Table 4:** Signal strength of the Preferred Term and the clinical priority assessing results.

System organ classes	Preferred term	Cases (*n*)	ROR (95% two-sided CI)	Death (*n*)	IMEs/DMEs	Relevant evidence evaluation	Priority level (score)
Eye disorders	Choroiditis	47	92.85 (69.26–124.49)	0	IME	−	Weak (4)
	Visual acuity reduced	54	60.02 (45.74–78.75)	0	NA	++	Moderate (6)
	Mydriasis	40	59.34 (43.34–81.25)	0	NA	++	Moderate (5)
	Uveitis	47	57.41 (42.93–76.76)	0	IME	−	Weak (4)
	Eye pain	64	42.27 (32.9–54.29)	0	NA	++	Moderate (6)
	Choroidal detachment	10	20.72 (11.1–38.67)	0	IME	−	Weak (4)
	Corneal oedema	7	14.18 (6.75–29.82)	0	NA	+	Weak (3)
	Photophobia	10	11.43 (6.14–21.28)	0	NA	++	Moderate (5)
	Vision blurred	22	8.64 (5.67–13.16)	0	NA	++	Moderate (5)
	Visual impairment	19	6.41 (4.08–10.08)	0	NA	++	Moderate (5)
	Eye irritation	9	8.17 (4.24–15.73)	0	NA	++	Weak (4)
	Eye discharge	6	9.79 (4.39–21.82)	0	NA	+	Weak (3)
	Iridocyclitis	5	10.11 (4.2–24.33)	0	IME	−	Weak (3)
	Ulcerative keratitis	5	10.1 (4.2–24.3)	0	IME	−	Weak (3)
	Strabismus	5	9.96 (4.14–23.96)	0	NA	+	Weak (3)
	Posterior capsule opacification	4	8.86 (3.32–23.67)	0	IME	−	Weak (3)
	Retinal detachment	4	6.98 (2.62–18.62)	0	IME	++	Moderate (5)
	Eye pruritus	5	5.33 (2.22–12.84)	0	NA	+	Weak (3)
	Anterior segment ischemia	3	6.99 (2.23–21.92)	0	IME	−	Weak (3)
	Ciliary hyperemia	3	6.98 (2.23–21.83)	0	NA	−	Weak (2)
	Lagophthalmos	3	6.92 (2.22–21.49)	0	NA	−	Weak (2)
	Corneal scar	3	6.88 (2.21–21.37)	0	NA	−	Weak (2)
	Ocular hyperemia	5	4.73 (1.97–11.39)	0	NA	++	Weak (3)
	Retinal Degeneration	3	6.71 (2.16–20.84)	0	IME	−	Weak (3)
	Blindness	5	4.67 (1.94–11.24)	0	DME	++	Moderate (5)
	Vitreous hemorrhage	3	6.41 (2.06–19.9)	0	IME	−	Weak (3)
	Photopsia	3	5.86 (1.89–18.18)	0	NA	++	Weak (4)
	Miosis	3	5.57 (1.79–17.29)	0	NA	−	Weak (2)
	Eye swelling	3	3.33 (1.07–10.32)	0	NA	++	Weak (3)
Cardiac disorders	Tachycardia	12	4.84 (2.74–8.55)	0	NA	++	Weak (4)
	Bradycardia	4	2.96 (1.11–7.89)	0	NA	−	Weak (1)
Gastrointestinal disorders	Large intestine perforation	8	13.36 (6.67–26.77)	0	IME	−	Weak (3)
	Intestinal obstruction	8	6.87 (3.43–13.76)	0	IME	−	Weak (3)
	Mouth swelling	4	7.29 (2.73–19.44)	0	NA	−	Weak (2)
	Impaired gastric emptying	3	4.58 (1.48–14.22)	0	IME	−	Weak (2)
	Swollen tongue	4	3.67 (1.37–9.78)	0	NA	++	Weak (3)
General disorders and administration site conditions	Thirst	9	11.65 (6.05–22.43)	0	NA	++	Weak (4)
	Condition aggravated	58	6.01 (4.62–7.81)	0	NA	−	Weak (4)
	Hyperthermia	4	6.88 (2.58–18.35)	0	NA	++	Weak (4)
	Feeling jittery	4	5.63 (2.11–15.03)	0	NA	++	Weak (4)
	Drug ineffective	42	1.85 (1.36–2.52)	0	NA	−	Weak (1)
Infections and infestations	Endophthalmitis	74	128.78 (101.89–162.75)	0	IME	−	Moderate (5)
	Hypopyon	4	8.8 (3.3–23.49)	0	IME	−	Weak (3)
	Septic shock	8	5.06 (2.53–10.14)	0	IME	−	Weak (3)
	Eye abscess	3	6.92 (2.23–21.5)	0	IME	+	Weak (4)
	Conjunctivitis	4	5.1 (1.91–13.61)	0	NA	++	Weak (4)
Injury, poisoning and procedural complications	Product packaging confusion	13	25.87 (14.97–44.69)	0	NA	−	Weak (3)
	Product label confusion	7	8.61 (4.1–18.09)	0	NA	−	Weak (2)
	Anesthetic complication neurological	4	8.83 (3.31–23.57)	0	IME	−	Weak (3)
	Product use issue	22	4.08 (2.68–6.21)	0	NA	−	Weak (2)
	Wrong product administered	4	7.49 (2.81–19.97)	0	NA	−	Weak (2)
	Product dispensing error	4	6.1 (2.29–16.28)	0	NA	−	Weak (2)
	Hyphema	3	6.86 (2.21–21.31)	0	IME	+	Weak (4)
	Incorrect route of product administration	4	3.33 (1.25–8.88)	0	NA	−	Weak (1)
	Toxicity to various agents	9	2.13 (1.11–4.1)	2	NA	++	Moderate (5)
Investigations	Intraocular pressure increased	50	75.49 (56.93–100.09)	0	NA	++	Moderate (5)
	Blood pressure systolic decreased	9	14.96 (7.77–28.82)	0	NA	−	Weak (2)
	Body temperature decreased	9	9.59 (4.98–18.47)	0	NA	−	Weak (2)
	Blood pressure systolic increased	9	6.31 (3.28–12.15)	0	NA	++	Weak (4)
Metabolism and nutrition disorders	Polydipsia	9	16.5 (8.57–31.78)	0	NA	++	Weak (4)
	Type 2 diabetes mellitus	9	3.06 (1.59–5.9)	0	IME	−	Weak (2)
Musculoskeletal and connective tissue disorders	Chondritis	4	8.93 (3.34–23.87)	0	NA	−	Weak (2)
Nervous system disorders	Anticholinergic syndrome	21	40.31 (26.19–62.05)	0	IME	++	Moderate (6)
	Coma	10	7.92 (4.25–14.76)	0	IME	++	Moderate (6)
	Hypotonia	6	9.7 (4.35–21.62)	0	NA	−	Weak (2)
	Pyramidal tract syndrome	4	8.88 (3.32–23.72)	0	IME	−	Weak (3)
	Somnolence	14	3.1 (1.83–5.24)	0	NA	++	Weak (4)
	Myoclonus	3	4.77 (1.54–14.8)	0	NA	++	Weak (3)
Product issues	Needle issue	9	10.85 (5.63–20.9)	0	NA	−	Weak (2)
Psychiatric disorders	Agitation	11	6.73 (3.72–12.18)	0	NA	++	Moderate (5)
	Hallucination, visual	6	7.19 (3.22–16.03)	0	IME	++	Moderate (5)
	Confusional state	15	2.79 (1.68–4.64)	0	NA	++	Weak (4)
	Hallucination	5	2.76 (1.15–6.65)	0	IME	++	Weak (4)
Renal and urinary disorders	Polyuria	9	14.01 (7.27–26.97)	0	NA	−	Weak (2)
	Pollakiuria	9	7.2 (3.74–13.86)	0	NA	−	Weak (2)
	Urinary retention	4	3.65 (1.37–9.75)	0	IME	++	Weak (4)
Skin and subcutaneous tissue disorders	Erythema	14	3.11 (1.84–5.26)	0	NA	++	Weak (4)
	Madarosis	4	5.62 (2.1–14.98)	0	NA	+	Weak (3)
	Toxic epidermal necrolysis	4	5.02 (1.88–13.38)	0	DME	−	Weak (4)
	Hyperhidrosis	9	3.11 (1.62–5.99)	0	NA	++	Weak (3)
Vascular disorders	Flushing	9	4.78 (2.48–9.2)	0	NA	++	Weak (3)
Missing or unknown	Wrong drug administered	9	16.49 (8.56–31.77)	0	NA	−	Weak (2)
	Drug effect prolonged	3	6.9 (2.22–21.46)	0	NA	−	Weak (2)

OR, reporting odds ratio; CI, confidence interval; IMEs, Important Medical Events; DMEs, Designated Medical Events; NA, Not Applicable (for relevant criteria); n, number of cases. ++: AEs are mainly from the FDA Prescribing Information, the Summary of Product Characteristics of posted by the MHRA, Phase 2/3 RCTs, or systematic reviews, with biological plausibility. +: AEs are mainly from other clinical trials, observational studies, or case reports/series with potential biological plausibility. -: AEs only emerging from disproportionality analyses.

## 4 Discussion

Ophthalmic atropine is a fundamental therapeutic agent in clinical ophthalmology, with its use notably expanding in pediatric myopia control (2, 3, 16). While its safety profile is generally considered favorable, a systematic, large-scale analysis of its associated adverse drug reactions (ADRs), particularly concerning rare but severe ocular events, remains lacking. Our study represents is the first comprehensive data mining analysis of the FAERS database focused specifically on ophthalmic atropine. We have systematically identified, quantified, and prioritized a spectrum of potential ADR signals, providing an evidence-based overview that extends beyond commonly recognized effects to include significant signals for ocular conditions such as endophthalmitis and intraocular pressure increased. It is worth noting that all findings should be interpreted as signals requiring confirmation rather than established causal relationships.

### 4.1 Potential clinical significance of ocular ADRs

Endophthalmitis emerged as the most strongly signaled ADR in our study, which was a finding that carries potential clinical implications, despite the lack of prior reports linking it to ophthalmic atropine (17). As a vision-threatening ophthalmic emergency, endophthalmitis is traditionally associated with intraocular surgery or trauma (18–20). Our study shows that the use of ophthalmic atropine, even via topical administration, may be significantly associated with this severe infectious event. Although according to the FAERS database, signals cannot definitively establish causality (11) and may be influenced by reporting biases (such as the greater likelihood of serious events being reported). This association warrants clinical attention. It is important for clinicians to be vigilant for potential infection risks when prescribing ophthalmic atropine, particularly for long-term use. Reinforcing patient education on the critical importance of aseptic administration techniques is essential.

Furthermore, the study detected a series of high-intensity signals related to ocular inflammation and its manifestations, including choroiditis, uveitis, and hyperemia. These inflammatory responses could potentially be attributed to impurities or preservatives remaining from the atropine preparation process, the drug acting as a hapten, or its vasodilatory effect on capillaries (21). Previous studies have indicated that a minority of patients experience allergic conjunctivitis and other related symptoms (3, 8, 9, 22). However, the potential possibility that atropine might induce or exacerbate intraocular inflammatory reactions in specific, susceptible individuals cannot be entirely ruled out. Additionally, it is important to note that atropine is a standard medication used in the treatment of anterior uveitis to prevent posterior synechiae. Therefore, some inflammatory signals may be confused with indications, where manifestations of the underlying disease being treated are reported as adverse reactions.

An increase in intraocular pressure (IOP) is a recognized risk associated with mydriatic agents. Atropine induces cycloplegia, which can narrow the anterior chamber angle, increase resistance within the trabecular meshwork, and obstruct aqueous humor outflow through Schlemm’s canal, ultimately leading to elevated IOP (23). Previous studies have confirmed that atropine, as a cycloplegic, can precipitate angle-closure glaucoma (24). However, whether the long-term use of atropine for myopia control in children leads to a sustained IOP increase and consequent glaucoma remains inconclusive. Most previous studies report no significant change in mean IOP among children using low-concentration atropine for myopia control (23, 25). Zhao et al. compared the efficacy and safety of 0.01% atropine with the control by enrolling seven randomized controlled trials. The mean difference of the IOP between the groups from two studies was −0.08 mmHg, which was not statistically significant (26). However, significant individual differences may exist, and clinically relevant IOP elevation can still occur in a subset of patients (27). Case reports have documented instances of acute, significant IOP spikes in children wearing orthokeratology lenses following the use of 0.125% atropine (28). Therefore, in clinical practice, the potential adverse effect of atropine on IOP warrants attention for myopic children with predisposing factors for IOP elevation (such as high myopia). This necessitates a baseline assessment prior to initiation of therapy and the implementation of regular IOP monitoring during treatment.

Visual acuity reduced and vision blurred were direct consequences of atropine’s cycloplegic effect, which impairs the eye’s accommodative function, and rank among the most frequently reported side effects in clinical practice. A meta-analysis indicated that the incidence of vision blurred during atropine therapy was 7.5% (48/636), with higher concentrations associated with an increased reporting rate of this symptom (29–31). Foo et al. found that part-time use of 1.0% atropine was also relatively well tolerated, with blurring of near vision reported in 0.6% of cases (32). Yam et al. found that different concentrations of atropine (0.05, 0.025, and 0.01%) all induced mydriasis under photopic conditions (by approximately 1.03 mm, 0.76 mm, and 0.49 mm, respectively) (4). It is noteworthy that even short-term use of low-concentration atropine (0.01%) can lead to significant mydriasis and a reduction in accommodative amplitude (4, 33).

In addition, eye pain, eye irritation, and photophobia were commonly reported adverse effects associated with ophthalmic atropine use. Previous studies indicated that 3.95% of children developed eye pain, eye irritation, or epiphora within 1 year of treatment with 0.5% atropine (34). In the two-year ATOM2 trial, eye irritation was reported in 1.2% of children in both the 0.5 and 0.01% atropine groups (35). A meta-analysis further identifies photophobia as the most frequently reported adverse reaction, with an incidence of 25.1% (205/816), and notes a positive correlation between atropine concentration and the rate of photophobia (4). There were complaints of discomfort (1.2%) in a retrospective review of part-time use of 1.0% atropine (31). In a one-week observation, Cooper et al. found that 0.05% atropine was associated with a higher incidence of discomfort in children, whereas the 0.01 and 0.02% concentrations showed lower and comparable adverse effect profiles (36).

### 4.2 Clinical prioritization of the disproportionality signals

The prioritization of safety signals in this study was achieved through the application of a rating scale to the analysis of disproportionality data, aiming to prevent unnecessary warnings. In our analysis, two AEs (blindness and toxic epidermal necrolysis) were classified as DMEs (37), representing rare but very serious drug-related AEs. Additionally, 26 AEs were classified as IMEs (38), representing serious AEs that may not be immediately life-threatening or are extremely rare. Our analysis identified four moderate-priority signals attaining the highest score of 6 (visual acuity reduced, eye pain, anticholinergic syndrome, and coma). In this framework, moderate priority signals indicate events with stronger statistical associations and greater biological plausibility, suggesting increased clinical awareness and potential regulatory follow-up. And weak priority signals represent adverse events with elevated reporting frequencies but limited statistical robustness (e.g., few cases, wide confidence intervals), warranting periodic monitoring rather than immediate action. It is worth noting that this classification is intended to guide future research, not to imply confirmed causality. These findings collectively delineate two principal risk dimensions for ophthalmic atropine: local ocular injury and systemic anticholinergic toxicity. The strong level of supporting evidence (“++”) associated with several of these signals, including blindness and anticholinergic syndrome, reinforces the clinical relevance of these associations. These observed signals suggest that, in addition to transient local effects, there may be potential associations with serious outcomes such as permanent visual impairment and life-threatening systemic reactions. This finding warrants further investigation in better-designed studies to clarify any potential risks.

Our study confirmed that topical administration in the eye may can still lead to potential systemic absorption, resulting in adverse reactions such as tachycardia, thirst, polydipsia, and agitation, which maybe were characteristic of anticholinergic syndrome. Previous studies have indicated that atropine (0.5 mg) exerts a marked inhibitory effect on exocrine glands, including the salivary, sweat, lacrimal, and respiratory glands (39). Case reports have documented instances where the misuse of a higher concentration (1% atropine) or failure to compress the nasolacrimal duct after administration led to systemic symptoms. And these symptoms resolved following appropriate intervention (40). Furthermore, clinical trials lasting approximately 1 year have reported that 1% or 0.5% atropine can induce eye pain, headache, dry mouth, facial flushing, and elevated body temperature, with participants generally tolerating these reactions (33, 41). Improper administration or excessive dosing of ophthalmic atropine can lead to systemic absorption via the nasal mucosa, potentially resulting in anticholinergic adverse effects (42). To minimize this risk, clinicians should instruct patients to apply pressure to the inner canthus (nasolacrimal duct occlusion) for a few minutes after instillation, a simple maneuver that may help reduce systemic absorption and associated symptoms.

A cautious interpretation is warranted for severe events such as endophthalmitis, blindness, toxic epidermal necrolysis, and coma. Although biologically plausible, these signals lack supporting clinical details, such as information on concomitant procedures, co-medications, or underlying conditions, that are essential for assessing causality. Without access to full case narratives, it remains unclear whether these events are directly attributable to atropine or reflect alternative explanations. Therefore, these findings should be viewed as hypothesis-generating and warrant confirmation in future studies with access to granular patient-level data.

### 4.3 Limitations

Several limitations inherent to the FAERS database and disproportionality analysis methodology should be considered when interpreting our findings. First, as a spontaneous reporting system, the FAERS database is subject to potential under-reporting, reporting bias, and variable data quality, which may affect the completeness and representativeness of the identified signals. Second, the lack of a defined patient denominator precludes the calculation of true incidence rates for the adverse events associated with ophthalmic atropine. Third, disproportionality analysis can only identify statistical associations and cannot establish a definitive causal relationship between drug exposure and adverse outcomes. Unmeasured or residual confounding factors, such as specific dosing regimens, treatment duration, concomitant medications, and underlying patient comorbidities, could not be fully accounted for in this analysis. Fourth, our signal detection was based on reported adverse event pairs, and the clinical details or outcomes for individual patients were limited. Notably, the FAERS database lacks granular information on dose or concentration of atropine used, and does not allow for distinction between different formulations (e.g., compounded versus commercial products), which may have different safety profiles. Fifth, despite applying rigorous duplicate detection algorithms, the potential for duplicate or misattributed reports cannot be completely eliminated, which may introduce residual noise into the analysis. Finally, while this study provides a comprehensive signal spectrum, further clinical, epidemiological, and mechanistic studies are required to validate these signals, quantify their absolute risk, and elucidate the underlying pathophysiological processes.

## 5 Conclusion

In summary, this pharmacovigilance study, based on data mining of the FAERS database, identified multiple significant adverse reaction signals associated with ophthalmic atropine. Signals for ocular conditions, including endophthalmitis, choroiditis, and elevated intraocular pressure, were detected with notable strength and frequency. These findings confirm that ocular atropine use carries a potential burden of ocular system adverse drug reactions, necessitating vigilant monitoring in clinical practice. The results provide statistical evidence to support risk awareness among clinicians, guide patient monitoring strategies, and inform clinical decision-making. Owing to the inherent limitations of the FAERS database, including the lack of access to original clinical records, case narratives, and detailed information on dosing, timing, co-medications, surgical procedures, and underlying conditions. These findings should be interpreted as hypothesis-generating signals rather than confirmatory evidence of causal associations. Further clinical and epidemiological studies are required to confirm these safety signals, clarify risk factors, and establish effective risk minimization measures for patients requiring ophthalmic atropine therapy.

## Data Availability

The original contributions presented in the study are included in the article/Supplementary material, further inquiries can be directed to the corresponding author/s.

## Footnotes

1 In the FAERS database: PRIMARYID: Unique identifier for each report version, concatenated from Case ID and Case Version Number. CASEID: Number for identifying a FAERS case. CASEVERSION: Safety Report Version Number (1 for initial report, incremented for follow-ups). FDA_DT: Date FDA received Case. In subsequent versions of a case, the latest manufacturer received date will be provided (YYYYMMDD format).

2 In the FAERS database: Other Serious (Important Medical Events): Report when the event does not fit the other outcomes, but the event may jeopardize the patient and may require medical or surgical intervention (treatment) to prevent one of the other outcomes. Examples include allergic brochospasm (a serious problem with breathing) requiring treatment in an emergency room, serious blood dyscrasias (blood disorders) or seizures/convulsions that do not result in hospitalization. The development of drug dependence or drug abuse would also be examples of important medical events.
